# A novel magnet-based scratch method for standardisation of wound-healing assays

**DOI:** 10.1038/s41598-019-48930-7

**Published:** 2019-09-02

**Authors:** M. Fenu, T. Bettermann, C. Vogl, N. Darwish-Miranda, J. Schramel, F. Jenner, I. Ribitsch

**Affiliations:** 1 University of Veterinary Medicine Vienna, Department of Companion Animals and Horses, Equine Surgery Unit, VETERM, Veterinaerplatz 1, 1210 Vienna, Austria; 2 University of Veterinary Medicine Vienna, Department of Biomedical Sciences, Institute of Animal Breeding and Genetics, Veterinaerplatz 1, 1210 Vienna, Austria; 3 IST Austria, Bioimaging Facility, AM Campus 1, 3400 Klosterneuburg, Austria

**Keywords:** Collective cell migration, Biological models

## Abstract

A novel magnetic scratch method achieves repeatability, reproducibility and geometric control greater than pipette scratch assays and closely approximating the precision of cell exclusion assays while inducing the cell injury inherently necessary for wound healing assays. The magnetic scratch is affordable, easily implemented and standardisable and thus may contribute toward better comparability of data generated in different studies and laboratories.

## Introduction

Cell migration is fundamental to establishing and maintaining the proper organization of multicellular organisms and plays a pivotal role in numerous physiological and pathological processes including tissue morphogenesis and homeostasis, wound healing, immune surveillance, inflammation and cancer^1,2^. Due to its relevance to health and disease, methods to simulate and explore critical mechanisms of action and to investigate therapeutics under well-defined conditions are of broad interest, and a variety of *in vitro* models have been developed, of which the scratch assay is the most widely used^3,4^. The scratch assay, also known as wound healing assay, is implemented by creating a cell-free area (gap, wound) within a confluent monolayer either by removing the cells post adherence via mechanical, electrical, chemical, optical or thermal means, or through physical exclusion of cells during seeding^1–3,5–11^. Subsequently serial high-resolution images are captured and analysed to quantify the dynamics of cell migration into the gap^3^. However, as the scratch is most commonly created manually using a pipette tip, the extent of the cellular injury and the geometry of the scratch is influenced not only by the scraping tool utilized, but also the level of manual control, the pressure exerted, the angle of the instrument and the velocity of scraping^3,4,6,7^. As a result, repeatability and reproducibility of the assays is limited and dependent on the manual dexterity of the researchers. Variation during wounding may confound interpretation and quantification of the wound healing process and impede comparison of results amongst different researchers and laboratories^1,3,4,6,7^.

Therefore, we aimed to develop and establish a novel scratch method allowing creation of a consistent, reproducible scratch with relatively smooth edges and little cellular debris within the gap, that is easy to standardize across different operators, studies and even laboratories.

The novel scratch assay employs magnetic discs of standardised size, which are placed in the cell culture wells. The well-plate is then placed on a matching well-plate lid with affixed magnetic spherical counterparts. The magnetic force between the magnetic spheres and discs provides a standardized pressure and keeps the magnetic discs stationary in the wells while the plate is moved horizontally to create scratches of uniform size in all wells simultaneously. The new magnetic scratching technique is easy to adapt to well-plates of different sizes and shapes and can be handcrafted in every lab using standard cell culture well-plates and commercially available magnets.

## Results and Discussion

**Figure 1:**
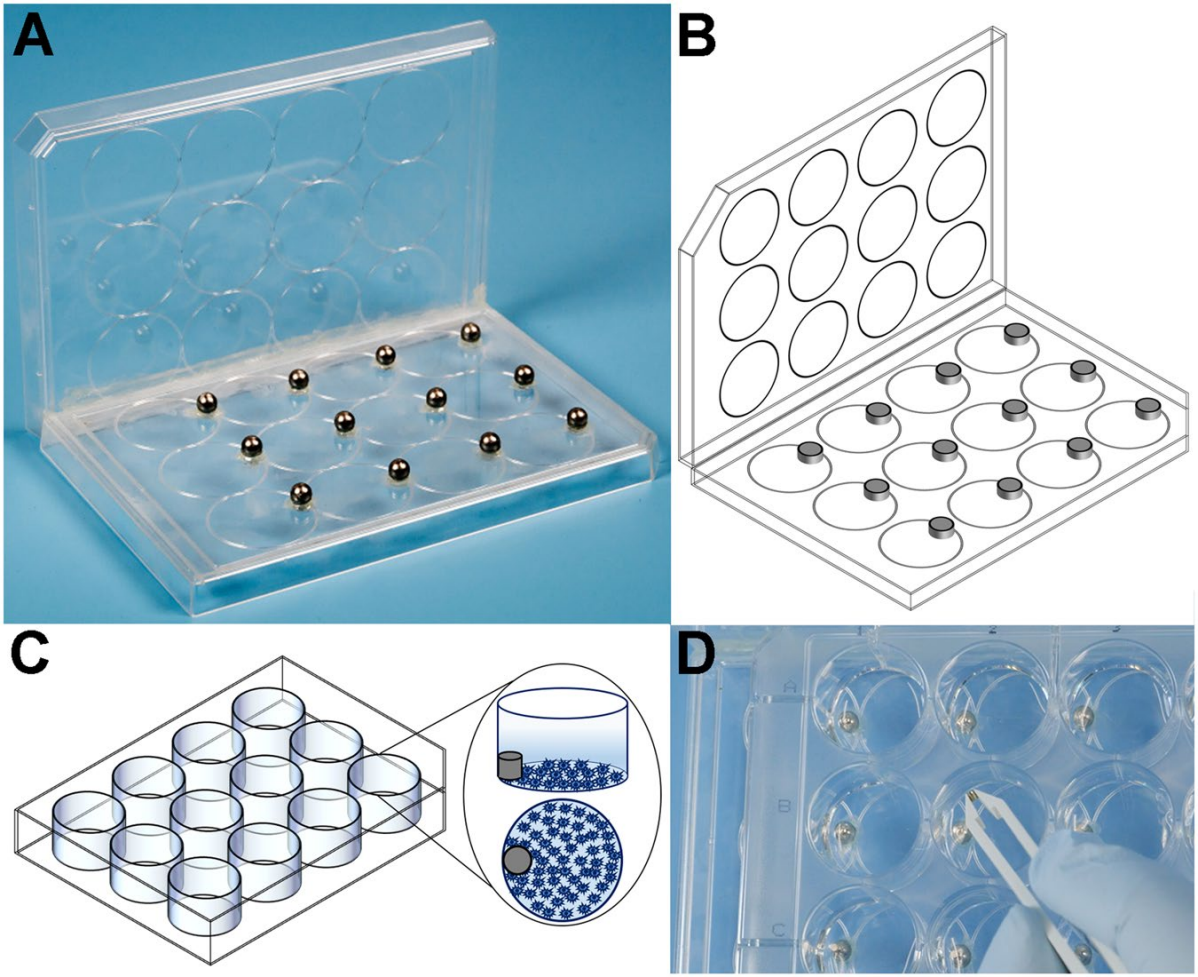
Figures (**A**) (photograph) and (**B**) (schematic) demonstrate the lid with the glued-on magnets on the right side of each etched-in well and the guide rail of the novel magnet scratch method, while figures (**C**) (schematic) and D (photograph) show the well-plate with the magnet being placed in the left side of each well.

**Figure 2:**
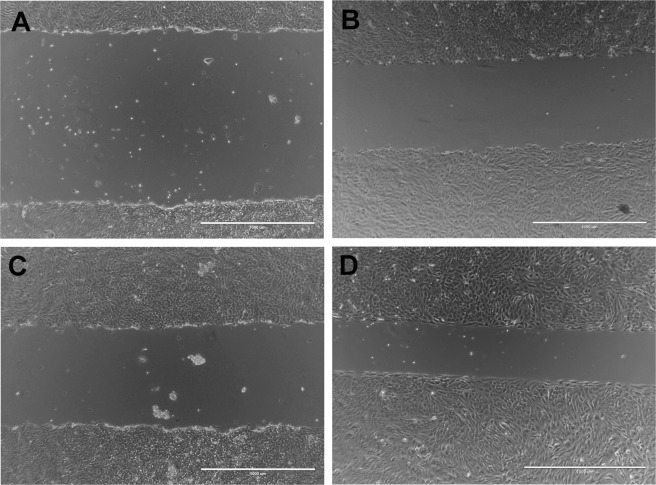
Phase contrast images of representative gaps created in confluent chondrocyte cultures using the (**A**) novel magnetic scratch method, (**B**) pipette tip (1250 µl) with 50 g manual pressure, (**C**) pipette tip (1250 µl) with 150 g manual pressure and (**D**) commercially available cell culture insert, at 0 h (scale bar = 1000 µm). Note differences in gap width homogeneity and gap border straightness between techniques.

To validate the novel magnet scratching technique, scratch assays were carried out in technical duplicates by 2 separate operators on confluent monolayers of primary equine tenocytes (n = 3 donors) and chondrocytes (n = 3 donors) employing one of four techniques: (1) the novel magnetic scratch method using 1.5 mm diameter magnetic discs (Fig. 1), (2) a commercially available cell culture insert for physical exclusion of cells in a defined area, (3) a scratch assay using a pipette tip (1250 µl) with 50 g and 4) with 150 g manual pressure (Fig. 2). The different techniques were compared using serial micrographs taken until full closure of the gap to evaluate the target variables I) standard deviation (SD) and coefficient of variation (CV) of the gap width measured at ten points along the gap, II); straightness of the gap margins (ratio of the measured length of the gap margins to the length of an ideal straight line) and III) gap closure rate (slope of the regression of gap area in mm^2^ depending on time) (Suppl. Figs 1 and 2).

Reproducibility and repeatability of the assays was determined based on the differences in gap width standard deviation and coefficient of variation observed between and within the 2 different operators (Table 1). The pipette 50 g technique had the worst repeatability and reproducibility (SD 113.8 vs. 63.19, CV 17.4% vs.7.89%), followed by the pipette 150 g (SD 71.16 vs. 59.62, CV 9.08% vs. 7.2%). The magnet (SD 28.72 vs. 33.18, CV 1.86% vs. 2.16%), and the insert (SD 12.2 vs. 24.85, CV 2.76% vs. 5.27%) had the best repeatability and reproducibility.

**Table 1:** Descriptive statistics of the gap width and the straightness of the gap margins achieved with the 4 scratch methods (pipette 50 g, pipette 150 g, magnet, cell exclusion insert) by the 2 operators as well as overall.

	Gap Width											
	pipette 50 g			pipette 150 g			magnet			Insert		
	OP1	OP2	both OPs	OP1	OP2	both OPs	OP1	OP2	both OPs	OP1	OP2	both OPs
Mean	654	800.7	728	784	828.2	798.1	1545	1533	1539	442.7	472	457.6
Std. Deviation	113.8	63.19	58.5	71.16	59.62	35.05	28.72	33.18	23.69	12.2	24.85	13.27
Std. Error of Mean	17.99	9.99	9.25	11.25	9.427	5.409	4.542	5.246	3.746	1.93	3.929	2.098
Lower 95% CI	617.6	780.5	709.3	761.2	809.1	787.2	1536	1523	1532	438.8	464	453.3
Upper 95% CI	690.4	821	746.7	806.8	847.2	809	1554	1544	1547	446.6	479.9	461.8
Coeff. of var.	17.4%	7.89%	8.04%	9.08%	7.20%	4.39%	1.86%	2.16%	1.54%	2.76%	5.27%	2.90%
	**Line Straightness**											
	**pipette 50 g**			**pipette 150 g**			**magnet**			**Insert**		
	**OP1**	**OP2**	**both OPs**	**OP1**	**OP2**	**both OPs**	**OP1**	**OP2**	**both OPs**	**OP1**	**OP2**	**both OPs**
Mean	1.25	1.25	1.24	1.22	1.20	1.21	1.14	1.14	1.13	1.07	1.07	1.07
Std. Deviation	0.08	0.06	0.06	0.07	0.04	0.06	0.07	0.06	0.05	0.04	0.04	0.04
Std. Error of Mean	0.02	0.02	0.01	0.02	0.01	0.01	0.02	0.02	0.01	0.01	0.01	0.01
Lower 95% CI	1.20	1.21	1.21	1.17	1.18	1.19	1.10	1.10	1.11	1.05	1.04	1.05
Upper 95% CI	1.30	1.28	1.27	1.27	1.23	1.24	1.19	1.17	1.15	1.09	1.09	1.09
Coeff. of var.	6.65%	4.51%	4.89%	5.85%	3.31%	4.77%	5.86%	4.95%	4.06%	3.40%	3.95%	3.61%

Scientific discovery is an iterative process and requires reproducible data, which allow multiple data sets to be combined to generate knowledge. Therefore, the need for scientific community-wide implementation of standardized assays to ensure that the data produced has high intra-study consistency and can be replicated and compared successfully across multiple laboratories is increasingly recognized. However, this can only be achieved with cost-effective, platform-independent, easy-to-use techniques, which can be implemented in any laboratory without technical prerequisites, such as the novel magnetic scratch or cell exclusion assays.

**Figure 3:**
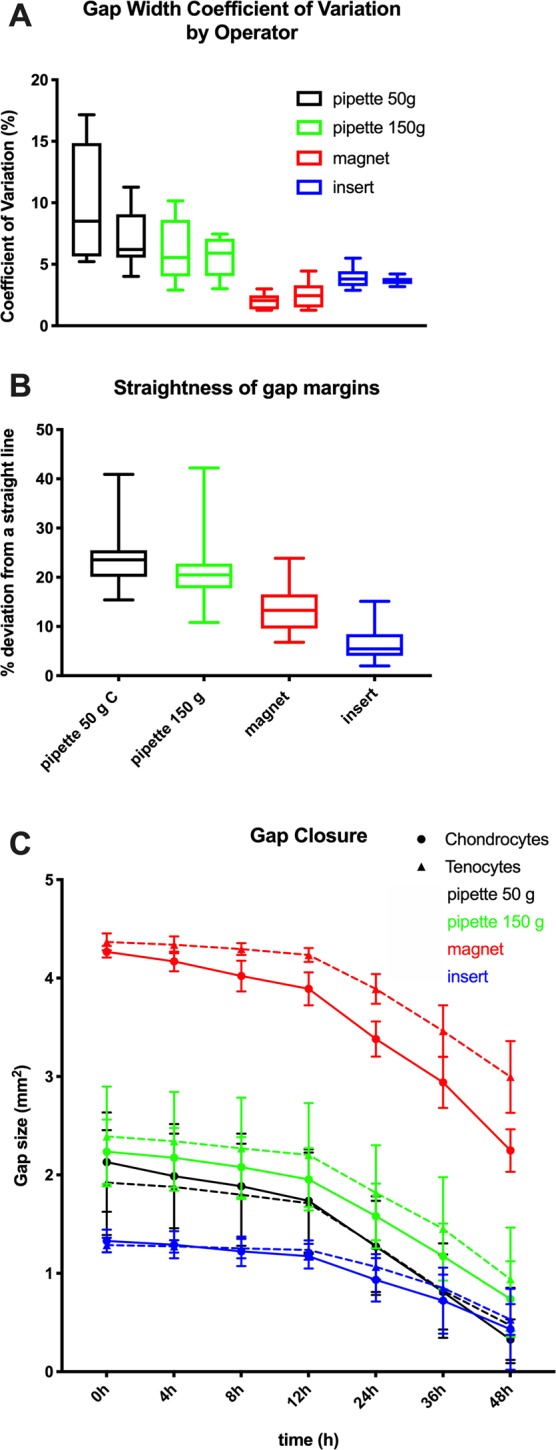
Comparison of the gap width coefficient of variation by operator, the straightness of the wound margins and the gap closure rate between the 4 scratch techniques. (**A**) Box and Whiskers plot of the gap width’s coefficient of variation in monolayer cultures. The left plot of each equally coloured pair corresponds to operator 1, the right to operator 2. The box extends from the 25th to 75th percentiles. The line in the middle of the box is plotted at the median. The whiskers indicate the minimum and maximum. Gap width varied least with the insert followed by the magnet, then the pipette 150 g and last the pipette 50 g. (**B**) Box and Whiskers plot of the straightness of the gap margins achieved in monolayer cultures of chondrocyte and tenocyte. The box extends from the 25th to 75th percentiles. The line in the middle of the box is plotted at the median. The whiskers indicate the minimum and maximum. Gap margins were straightest with the insert followed by the magnet, then the pipette 150 g and last the pipette 50 g. (**C**) The gap closure rate of chondrocytes and tenocytes from 0–48 hours, the time period of linear gap closure rate in both cell types before the gap closed in any sample, showing the mean remaining gap size ± SD for each time point. Cell type (chondrocyte versus tenocyte) had a significant influence only on gap closure rate (p < 0.001). With the scratch methods, which cause cell injury (magnet, pipettes), gap closure speed was faster than with the insert.

Gap width standard deviation varied significantly (p < 0.001) between methods (Table 1, Suppl. Table 1, Suppl. Fig. 3A). The insert (SD 13.27), followed closely by the magnet (SD 23.69), had the lowest standard deviation of the gap width, and the manual methods had the highest (50 g > 150 g, SD 58.5 resp. 35.05). The coefficient of variation of the gap width was smallest for the magnet with 1.54%, followed by the cell culture insert with 2.9%, the pipette 150 g with 4.39%, and the pipette 50 g with 8.04% (Fig. 3A, Suppl. Fig. 3B). Correspondingly, straightness of the gap margins varied significantly (p < 0.001) between the cell exclusion insert and the other three methods as well as between the magnet and the other three methods (Fig. 3B, Table 1, Suppl. Table 1, Suppl. Fig. 3C). Cell exclusion inserts led to the straightest margins (mean deviation from the ideal straight line = 6.9%, CV = 3.61%), the magnet was intermediate (mean deviation from the ideal straight line = 13.2%, CV = 4.06%), and the pipette methods (50 g > 150 g) showed the largest difference (mean deviation from the ideal straight line = 23.9% resp. 21.1%, CV = 4.89% resp. 4.77%) between the measured and an ideal margin (of a perfect straight line) (Table 1, Suppl. Table 1).

These results highlight the significantly higher geometrical control and precision of the cell culture insert and the magnet in comparison with the manual techniques.

Two cell types (chondrocytes and tenocytes) were included in the validation study of the magnetic scratch to determine whether cell type would, due to differences in cell-cell adhesion, polarity or cytoskeletal organisation, influence gap linearity, gap width SD and CV and gap closure rate. In contrast to chondrocytes, tenocytes have long cell processes and adherens and gap junction-based cell-to-cell contacts forming complex interconnected networks and cell sheets^12,13^. The gap junctions are essential for cell-cell communication and represent a functional network allowing coordination of mechanical and synthetic activity^12,13^. However, cell type showed a significant influence only on gap closure rate (p < 0.001) but not on any indicator of gap geometry, accuracy or precision.

Gap closure rate also differed significantly (p < 0.001) among methods (Fig. 3C, Table 1, Suppl. Table 2, Suppl. Fig. 3D) and was significantly slower (p < 0.001) with the cell exclusion than the injury inducing scratch methods for both cell types as well as overall. This is consistent with previous studies showing differences in cell migration and signalling between cell exclusion and cell injury wound healing assays.

**Figure 4:**
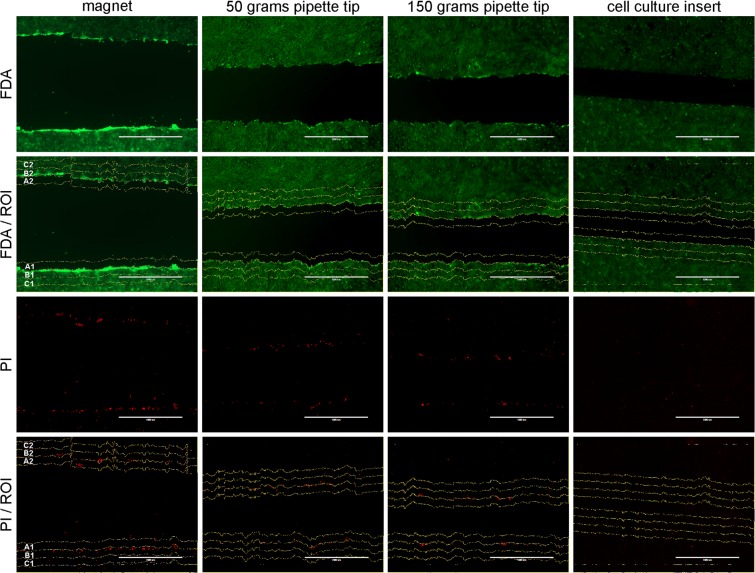
Definition of regions of interest (ROI) along the scratch margins. ROI (**A**) extends 25 µm from the scratch margin into the cell free area of the scratch; ROI (**B**) extends 25 µm from the scratch margin into the remaining cell monolayer (region of main interest: contains cells which died in direct consequence to wounding); ROI (**C**) extends from 25 µm to 50 µm into the remaining cell monolayer (scale bar = 1000 µm).

In this study, Fluoresceindiacetat (FDA, C-7521, Sigma-Aldrich) - Propidiumiodid (PI, P4170, Sigma-Aldrich) fluorescent staining confirmed the accumulation of dead cells alongside the scratch gap border for all techniques except the cell culture insert (Fig. 4). The difference in the number of dead cells between all three injury-inducing techniques (magnet, pipette 150 g pipette 50 g) and the cell culture insert was statistically significant (p < 0.001, Suppl. Table 3) at all three time points of analysis (0 h, 8 h and 24 h after wounding) independent of cell type (chondrocytes versus tenocytes, Suppl. Table 4, Suppl. Fig. 4). While the injury free release of contact inhibition is sufficient to induce cell migration in cell exclusion assays^2,6,9,14^, the injury inflicted upon the cells in scratch wound healing models with the associated disruption of cell adhesions and release of intracellular contents and signalling molecules has been shown to alter the microenvironment in the cell culture and to provide a critical input required for cell-cell coordination in the spreading of the wounded monolayer^1,3,4,6,7,9^. Thus, while cell exclusion assays provide repeatability and geometric control, they fail to induce an injury and the insert used in this study correspondingly led to slower gap closure kinetics compared to the three assays that inflicted injury upon the cell layer.

Gap closure rate typically shows a nonlinear decline with decreasing gap size^15^, which we could confirm for all techniques used in this study (Suppl. Fig. 3D). For small gaps this limits the time period in which linear closure rates allow comparative quantification of cell migration to 48 hours. As the scratch assay is predominantly used to study wound healing and cancer cell migration and for corresponding drug discovery screening^7^, the creation of a larger standardized gap, as introduced in this study (mean gap width 1539 μm (magnet) versus 457.6 μm (cell culture insert), Table 1), provides a longer observation period to study, e.g., the tumour cell aggressiveness or the efficacy and cytotoxicity of the screened molecules and compounds.

As damage to the plate surface incurred during scratching may affect migration rates^1,8^, the surface relief of the culture dish was measured using atomic force microscopy (Suppl. Fig. 5). The unscratched surface was characterized by topographical variation within 40 nm in height. Pipette tip scratches with 150-gram pressure increased topographical variation to 160 nm, and the magnetic scratch method to 250 nm, both with grooves parallel to the orientation of the scratch. However, this level of variation in surface topography appears to be no impediment to cell migration since the gap closure rates of the three different injury-inducing scratch methods were comparable and faster than the cell exclusion assay.

In conclusion, the novel magnetic scratch method combines operational ease with precision and geometric control, induces the cell injury inherently necessary for a wound healing assay and provides excellent repeatability, reproducibility and easy standardization across different observers (Table 2). Thus, the proposed method may contribute to the generation of data, which are better comparable between different studies and laboratories, and may lead to more efficacy and quality control in research.

**Table 2:** Summary of advantages and disadvantages of different wounding methods.

	Manual scratching		Cell culture insert		Magnet	
	Advantage	Disadvantage	Advantage	Disadvantage	Advantage	Disadvantage
Margin straight-ness		Varies within and between operators	Very good		Good	
Gap width		Varies within and between operators	Standardized	Narrow	Smallest coefficient of variation; Wider than other techniques: Longer time span until gap closure = longer observation period. Assays of longer duration might more accurately reflect the combined effects of migration and proliferation^10^	
Homo-geneity		Varies within and between operators	Standardized		Good	
Repeat-ability/Reproduc-ibility		Variation within and between operators	Very good		Good	
Damage to the plate surface	Little		None			Possible
Adapt-ability to plate size	Good			Only one size available	Good	
Simult. scratching of more than one well		Not possible	N.A.	N.A.	Possible	
Price	Lowest costs			Expensive	Very low costs	
Avail-ability	Always and immediately			Needs to be ordered, available in packages of limited numbers.	All parts to tailor scratching device (but the magnets) are standard laboratory equipment.	Magnets need to be ordered. Requires some time to tailor the device.
Other challenges		Steady hand required upon scratching				Steady hand required upon scratching.
Sum	4	6	5	4	8	4

## Methods

### Production and handling of the magnetic scratching tool

The novel magnetic scratching tool can be handcrafted easily by using standard cell culture well-plates and commercially available neodymium magnets. It can be tailored for different types of well-plates and used to scratch all wells of a plate simultaneously. First, the lids of two well-plates were glued together in a 90° angle along their long sides (Fig. 1) with a commercially available standard super glue, such that one lid serves as bottom plate and one as guiding rail. Then magnetic spheres (neodymium magnets, diameter 5 mm+/−0,1 mm, type K-05-C, Supermagnete, Germany) were glued to the lid serving as bottom plate, which should match the well-plate to be scratched and have the size and shape of the wells engraved as template to facilitate correct placement. The magnetic spheres were glued centred to the far-right end of each engraved well with vertically oriented magnetic force (Fig. 1) while using additional magnetic spheres as counter-parts to enable magnetic alignment.

The magnets used as scratching tools were disc-shaped (Supermagnete, S-1.5-0.5-N) with a diameter of 1,5 mm and height of 0,5 mm (+/−0,1 mm tolerance). Our pilot experiments determined four stacked discs to produce the optimal weight and magnetic force to create homogenous scratches over the full length of the wells. Prior to use, the discs were sterilized in 70% ethanol for 10 minutes followed by two washing steps with PBS for 5 minutes each. To position the scraping magnets, the cell culture well-plate was placed on top of the bottom plate offset to the right, such that the left rims of the wells overlapped the right rims of the engraved well shapes with the magnetic spheres on the bottom plate. The scratching magnets (stack of 4 discs) were dropped into the wells above the magnetic spheres using plastic tweezers. Due to the magnetic attraction they fell into place at the left rim of the wells directed toward the magnetic sphere on the bottom plate. When all magnets were in place the well-plate was shifted to the left, using the rail plate to facilitate creation of straight scratches, until it superimposed the bottom plate. Finally, the magnetic discs were removed from all wells using a sterile spherical magnet attached to a metal bar (Suppl. Video).

### Validation of the novel scratching tool

To validate the newly developed scratching tool, it was compared to the current gold standard (manual scratching with a pipette tip) and a cell culture insert for physical exclusion of cells in a defined area (80209, Ibidi^TM^). The validation study was carried out in 12-well cell culture plates and performed with equine chondrocytes and tenocytes (3 biological replicates each) by two independent operators to study repeatability and reproducibility of the different scratch assay methods.

The cells had been obtained from three horses, which were euthanized for reasons unrelated to this study. Tissue collection to obtain these cells had been performed according to the “Good Scientific Practice and Ethics in Science and Research” regulation implemented at the University of Veterinary Medicine Vienna. The animal owner’s consent to collect and analyse the samples and to publish resulting data was obtained according to the standard procedure and approved by the ethics and animal welfare committee of the University of Veterinary Medicine Vienna. Cells were cultured at routine cell culture conditions using Dulbecco’s modified eagle medium (DMEM, LONBE12-707F, Lonza) supplemented with 10% fetal bovine serum (FBS, F7524, Sigma-Aldrich), 1% L-glutamine (K0302, Biochrom), 1% penicillin-streptomycin (P4333-100ML, Sigma Aldrich), and 1% amphotericin (A2612, Biochrom) prior to seeding them onto 12 well-plates for the scratch assays in passage 4–5. Medium was changed twice weekly.

For the three scratching tools (magnet, pipette 50 g and pipette 100 g) 100.000 cells were seeded per well. 24 hours later the confluent cell layers were washed twice with PBS and the wounding of the cell layer was performed:

For manual scratching a 1250 µl pipette tip was used applying two different pressures (50 g and 150 g). Application of the correct pressure was monitored by performing the scratching procedure on a precision lab scale. To achieve straight scratches a ruler was used as guiding device.

Scratching with the magnet device was performed as described above.

Linear cell culture inserts (80209, Ibidi^TM^) for physical exclusion of cells at a defined distance (500 µm +/−100 µm) were used according to the manufacturer´s instructions. In brief, the inserts were placed in the centre of each well. 15.000–20.000 cells were seeded into each of the two insert chambers to achieve confluence the following day. To create the gap, the insert was removed using a sterile forceps.

Independent of the scratching technique the cells were washed twice with PBS following gap creation prior to adding new culture medium.

### Comparison of the cell free gaps (scratches)

The cell free gaps were imaged in phase contrast using the EVOS FL Auto imaging system with a 4x fluorite objective (ThermoFisher Scientific, AMEP4680). To ensure that pictures were always taken at the same position, the “reuse settings” function was applied, which allows resuming of previously used coordinates. Pictures were taken at 0, 4, 8, 12 hours and then every 12 hours until full gap closure (Suppl. Fig. 2). The size of the gaps was measured at all time points using the MRI Wound healing tool (http://dev.mri.cnrs.fr/projects/imagej-macros/wiki/Wound_Healing_Tool) in ImageJ (https://imagej.nih.gov/ij/, version 2.0.0-rc-43/1.50e). The micrographs taken at 0 hours were additionally analysed for straightness of the wound margins and for homogeneity of the gap width (Suppl. Fig. 1).

#### Homogeneity of the gap width

Gap homogeneity was evaluated by the mean, standard deviation (SD), and coefficient of variation (CV = SD/mean) of 10 measurements of the gap width performed at equal distances of 128 pixels (Suppl. Fig. 1) using ImageJ (version 2.0.0-rc—43/1.50e, Straight Hand Line Tool, NIH, USA) to measure the distance between the upper and lower gap margins.

#### Straightness of the gap margins

The margins of the gaps were evaluated relative to an ideal (straight) line, parallel to the horizontal axis of the picture, with a length of 1280 pixel (Suppl. Fig. 1). The length of the gap was analysed with the Free Hand Line Tool on ImageJ version 2.0.0-rc—43/1.50e. Straightness of the gap margins, defined as the length of the edge of the gap divided by the length of a straight line was calculated as the average of the ratio between the length of each gap margin (upper and lower) and the ideal line (Straightness = (length of upper gap margin + length of bottom gap margin)/ (2*length of ideal line)).

#### Gap closure rate

Gap area was measured at successive time-points (0, 4, 8, 12, 24, 36, 48, 60, 72, 84 hours) using the MRI Wound healing Tool (http://dev.mri.cnrs.fr/projects/imagej-macros/wiki/Wound_Healing_Tool) on ImageJ version 2.0.0-rc—43/1.50e. The gap closure rate, defined as the slope of the regression of the gap area [mm^2^] depending on time, was recorded during the period of linear closure between 24 hours (after the initial lag period) and 48 hours (before the gap closed in any of the samples, Suppl. Fig. 1).

### Life-dead staining and analysis

To distinguish between life and dead cells a vital fluorescent double staining was performed using Fluoresceindiacetat (FDA, C-7521, Sigma-Aldrich) and Propidiumiodid (PI, P4170, Sigma-Aldrich) following manufacturer’s instructions. Based on the Esterase dependent transformation of non-fluorescent FDA into its fluorescent metabolite fluorescein, cells stained in green indicate life cells. PI was used as a counterstaining, to stain cell nuclei of dead cells. Subsequently, to evaluate the direct influence of the wounding method (scratching versus cell exclusion method) on the number of dead cells, FDA/PI stained cultures (n = 279, thereof n = 140 chondrocytes and 139 tenocytes) were imaged using the EVOS FL Auto imaging system (GFP EVOS LED light cube,AMEP4651, Emission 510/42 nm, Excitation 470/22 nm and Texas Red EVOS LED light cube, AMEP4655, Emission 628/32 nm, Excitation 585/29 nm) at 0 h, 8 h and 24 h after “scratching”and analysed using ImageJ. To distinguish between the overall occurrence of dead cells in the culture and cells, which died in direct consequence to wounding, three regions of interest (ROI) were defined along each scratch margin (lower and upper margin) for each micrograph (Fig. 4): (1) ROI (A) extends 25 µm from the scratch margin into the cell free area of the scratch; (2) ROI (B) extends 25 µm from the scratch margin into the remaining cell monolayer and is the region of main interest as it contains cells which died in direct consequence to scratching; and (3) ROI (C) extends from 25 µm to 50 µm into the remaining cell monolayer. The number of dead cells per ROI was counted manually and the dead cells per ROI of the lower (A1, B1, C1) and upper (A2, B2, C2) scratch margins were added to a total count per ROI. To interpret the influence of the wounding method on cell death and calculate the true amount of “scratch” related cell death per picture (relative number of dead cells in ROI(B)) the following formula was used $\frac{({\boldsymbol{B}}1+{\boldsymbol{B}}2)-({\boldsymbol{C}}1+{\boldsymbol{C}}2)}{({\boldsymbol{A}}1+{\boldsymbol{A}}2)}$. Negative values in the raw data resulting from a higher number of dead cells in ROI(C) than ROI(B) were set to zero.

### AFM

In order to assess potential damage to the plate surface due to scratching, the surface reliefs of wells scratched with the magnet scratch method and the pipette with 150-gram pressure were measured using atomic force microscopy and compared to a non-scratched well and a positive control (scratch performed using a #3 scalpel handle with 50 g pressure). Scratching was performed in wells containing PBS. After scratching, the wells were washed twice with ultrapure water to remove any precipitates of the PBS. The surface morphology was evaluated by Atomic Force Microscopy, using a Nanowizard 4 system from JPK (JPK Instruments AG, Berlin, Germany). The AC mode was used, with PPP-NCHAuD cantilevers (330 kHz nominal resonance frequency, 42 N/m force constant and 125microns length). Scan areas of 8 × 100 µm^2^ were imaged at 26 × 256 pixel resolution with the scanning direction perpendicular to the scratch direction. Consecutive scans were performed to achieve a scanned area with a final dimension of 8 × 2000 µm^2^. Three areas per scratch method were imaged. Images were analysed with JPK software and 3D plot using the 3D surface plot in ImageJ.

### Statistical analysis

Target variables are: straightness of the gap margins, defined as the length of the edge of the gap divided by the length of an ideal straight line; mean and standard deviation of the gap width measured at ten points along the gap; and the gap area in mm^2^. Gap area was measured at successive time-points and the gap closure rate (slope of the regression of gap area in mm^2^ depending on time) was recorded at the period of linear closure between 24 hours (after the initial lag period) and 48 hours (before the gap closed in any of the samples). Descriptive statistics, means, standard deviations and coefficients of variation were calculated for the target variables (straightness of the gap margins, homogeneity of the gap width, gap closure rate). With all these target variables, unifactorial ANOVAs were calculated separately with method as factor for each cell type. Both cell types were also combined in analyses, where only for the target variable “slope” cell-type was entered as an additional factor, since growth of chondrocytes and tenocytes differs. We additionally performed more complicated multifactorial ANOVAs with factors: method, operator, biological replicate, and method*operator, for chondrocytes and tenocytes separately. Both cell types were also combined in analyses, where cell-type was entered as an additional factor. With the more complicated ANOVA models, qualitatively and quantitatively similar results were obtained.

### Ethical approval and informed consent

Tissue collection to obtain the cells used in this study has been performed according to the “Good Scientific Practice and Ethics in Science and Research” regulation implemented at the University of Veterinary Medicine Vienna. The animal owner’s consent to collect and analyse the samples and to publish resulting data was obtained according to the standard procedure and approved by the ethics and animal welfare committee of the University of Veterinary Medicine Vienna.

## Supplementary information

Video of the novel magnet-based scratch method

Supplementary figures 1-5 and supplementary tables 1-4

## Data Availability

The datasets generated during and/or analysed during the current study are available from the corresponding author on reasonable request.
