# Supplementary material for: A novel magnet-based scratch method for standardisation of wound-healing assays
Source: Sci Rep. 2019 Sep 2;9:12625. doi: 10.1038/s41598-019-48930-7 (PMC6718675; doi:10.1038/s41598-019-48930-7)
Supplement: Supplementary file 2 — Supplementary figures 1-5 and supplementary tables 1-4 [file 41598_2019_48930_MOESM2_ESM.pdf]

A novel magnet-based scratch method for standardisation of wound-healing assays

M. Fenu[1], T. Bettermann[1], C.Vogl[2], N.Darwish-Miranda[3], J. Schramel[1], F. Jenner[1*+], I. Ribitsch[1+],

[+]shared last author

*Corresponding author

M. Fenu: Michele.Fenu@vetmeduni.ac.at

T. Bettermann: Tobias.Bettermann@vetmeduni.ac.at

C. Vogl: Claus.Vogl@vetmeduni.ac.at

N. Darwish-Miranda: Nasser.Darwish-Miranda@ist.ac.at

J. Schramel: Johannes.Schramel@vetmeduni.ac.at

F. Jenner: Florien.Jenner@vetmeduni.ac.at

I. Ribitsch: Iris.Ribitsch@vetmeduni.ac.at

1 University of Veterinary Medicine Vienna, Department of Companion Animals and Horses, Equine Surgery Unit, VETERM, Veterinaerplatz 1, 1210 Vienna, Austria

2 University of Veterinary Medicine Vienna, Department of Biomedical Sciences, Institute of Animal Breeding and Genetics, Veterinaerplatz 1, 1210 Vienna, Austria

3 IST Austria, Bioimaging Facility, AM Campus 1, 3400 Klosterneuburg, Austria

Supplementary Figure Legends

Supplementary Figure 1

Illustration of the analysis methods for scratch homogeneity (A), straightness of the gap margins (B) and gap size (C). Scratch homogeneity was measured using 10 vertical measurements of the gap width (w_i) performed at equally spaced distances of 128 pixels. Straightness of the gap margins was calculated as the average of the ratio between the measured length of each gap margin (upper (l_a) and lower (l_b)) and an ideal straight line (l_i) (straightness = (length of upper gap margin + length of bottom gap margin) /2*length of ideal line). Gap size was measured with the MRI Wound healing Tool (http://dev.mri.cnrs.fr/projects/imagej-macros/wiki/Wound_Healing_Tool) in ImageJ (version 2.0.0-rc—43/1.50e).

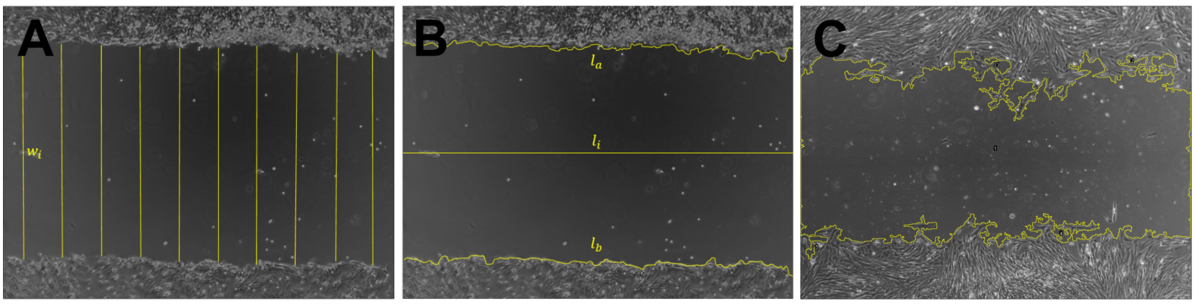

Supplementary Figure 2

Phase contrast images of representative gaps created in confluent chondrocyte (A) and tenocyte (B) cultures using the novel magnetic scratch method, the pipette tip (1250 µl) with 50g and 150g manual pressure and the commercially available cell culture insert, at 0h, 12h, 24h, 48h, and 84h (scale bar = 1000 µm). Note differences in gap size and closure time.

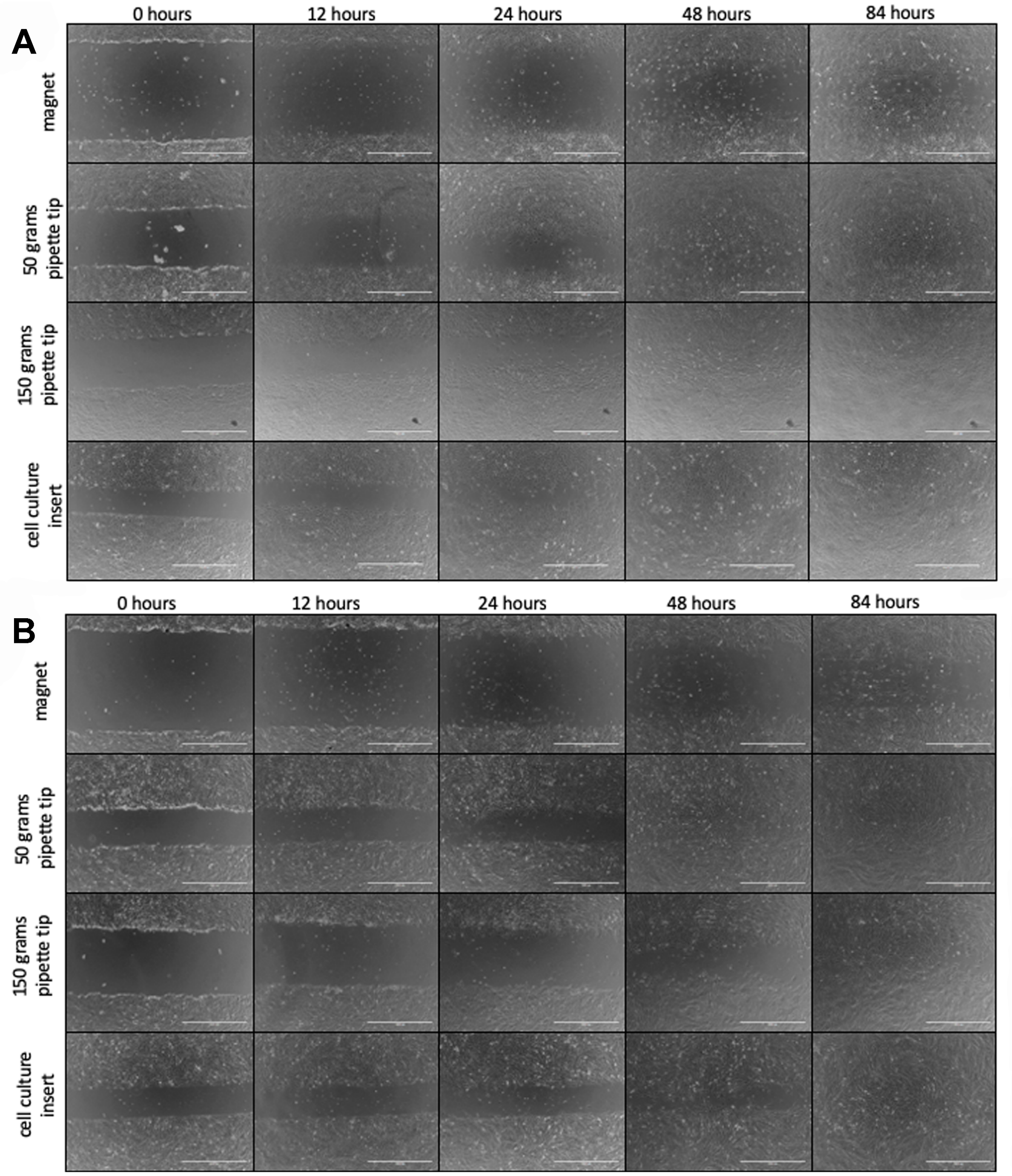

Supplementary Figure 3

A) Box and Whiskers plot of the gap width achieved in chondrocyte (A1) and tenocyte (A2) monolayer cultures by 2 different operators (operator 1 left, operator 2 right).The box extends from the 25th to 75th percentiles. The line in the middle of the box is plotted at the median. The whiskers indicate the minimum and maximum. As shown, the smallest variation in gap width was achieved using the insert followed by the magnet, the pipette 150g and last the pipette 50g.

B) Box and Whiskers plot of the gap width's coefficient of variation in chondrocyte (B1) and tenocyte (B2) monolayer cultures. The box extends from the 25th to 75th percentiles. The line in the middle of the box is plotted at the median. The whiskers indicate the minimum and maximum. As shown, the magnet and cell culture insert had the smallest coefficient of variation, the pipette 150g was intermediate and the pipette 50g had the highest coefficient of variation.

C) Box and Whiskers plot of the straightness of the gap margins achieved in chondrocyte (C1) and tenocyte (C2) monolayer cultures. The box extends from the 25th to 75th percentiles. The line in the middle of the box is plotted at the median. The whiskers indicate the minimum and maximum. As shown lines were straightest using the insert followed by the magnet and pipette 150g and last the pipette 50g.

D) Gap closure rate of chondrocytes (D1) respectively tenocytes (D2) throughout the observation period indicating the mean ± SD for each time point. As shown, the scratch methods which cause cell injury (magnet, pipettes) have a significantly faster gap closure rate than the cell exclusion method (insert). Closure speed was low with the insert, the scratch method with 150mg was intermediate, while the magnet and the scratch method with 50mg had the highest closure speeds. Gap closure rate appears to decrease when a gap size is small enough for contact inhibition to take effect.

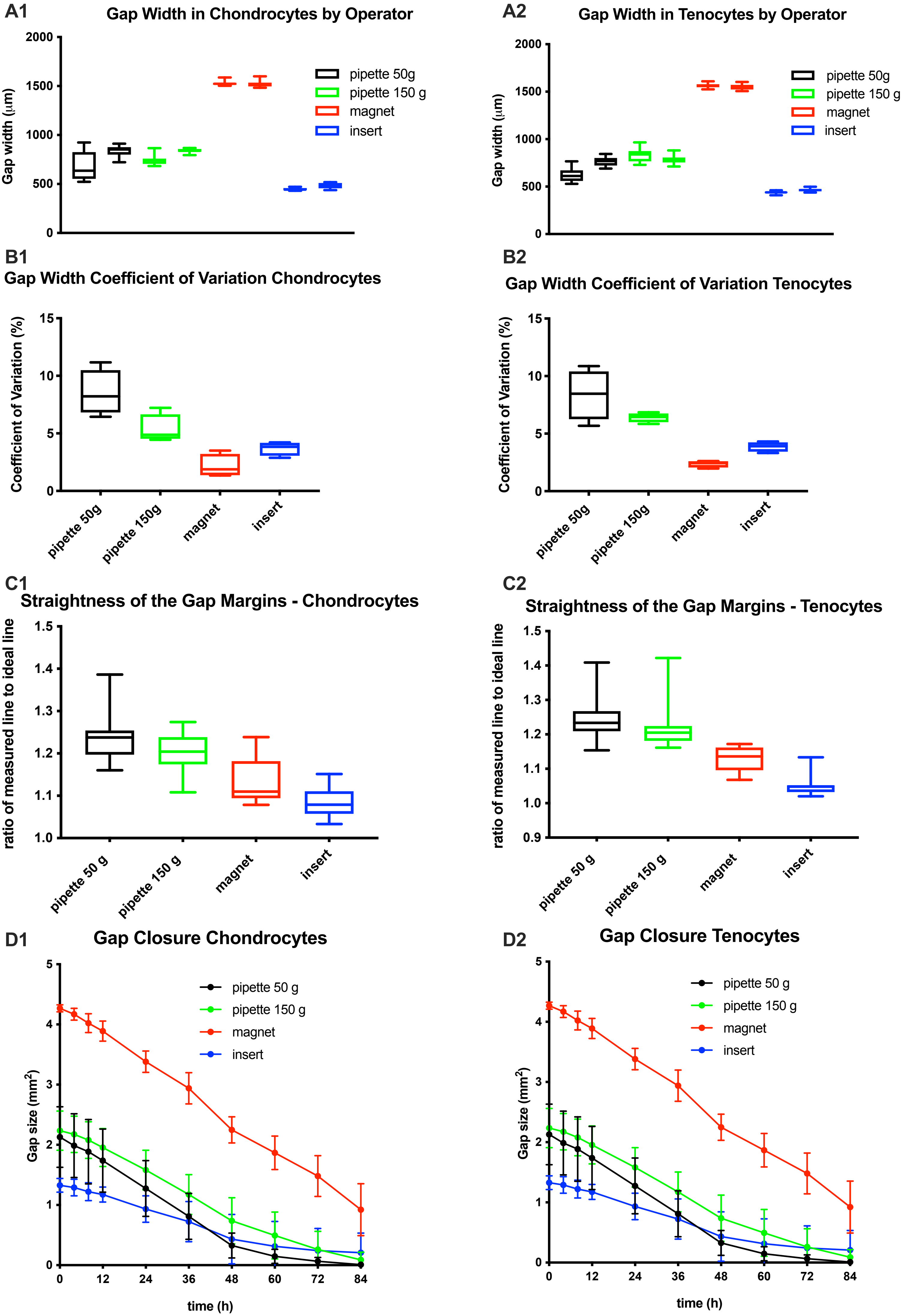
A1
Gap Width in Chondrocytes by Operator
Gap width (µm)
pipette 50g
pipette 150 g
magnet
insert
A2
Gap Width in Tenocytes by Operator
Gap width (µm)
pipette 50g
pipette 150 g
magnet
insert
B1
Gap Width Coefficient of Variation Chondrocytes
Coefficient of Variation (%)
pipette 50g
pipette 150g
magnet
insert
B2
Gap Width Coefficient of Variation Tenocytes
Coefficient of Variation (%)
pipette 50g
pipette 150g
magnet
insert
C1
Straightness of the Gap Margins - Chondrocytes
ratio of measured line to ideal line
pipette 50 g
pipette 150 g
magnet
insert
C2
Straightness of the Gap Margins - Tenocytes
ratio of measured line to ideal line
pipette 50 g
pipette 150 g
magnet
insert
D1
Gap Closure Chondrocytes
Gap size (mm²)
pipette 50 g
pipette 150 g
magnet
insert
time (h)
D2
Gap Closure Tenocytes
Gap size (mm²)
pipette 50 g
pipette 150 g
magnet
insert
time (h)

Supplementary Figure 4

Comparison of number of dead cells resulting from different wounding techniques (50g, 150g, magnet and cell culture insert) at 0h, 8h and 24h post injury for two cell types (Chondrocytes and Tenocytes). X-axis = wounding techniques at different timepoints for chondrocytes and tenocytes, Y-axis = number of cells per picture which died as a result of “scratching”.

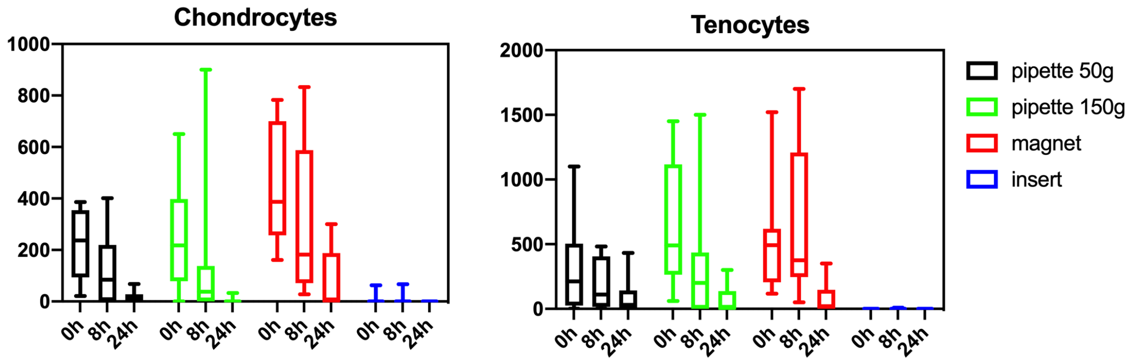

Supplementary Figure 5

Surface morphology of the scratched culture dishes was assessed by atomic force microscopy with a scan area of 8 x 100 µm^2. Depicted are a 3D view (top) and 2D view (bottom) of wells without scratch (A, negative control), scratched with (B) the magnet, (C) 150 grams pressure pipette tip and (D) a positive control (scratch performed using a #3 scalpel handle with 50g pressure). Please note the different scale used for the y-axis in images C and D to allow depiction of the extent of the damage.

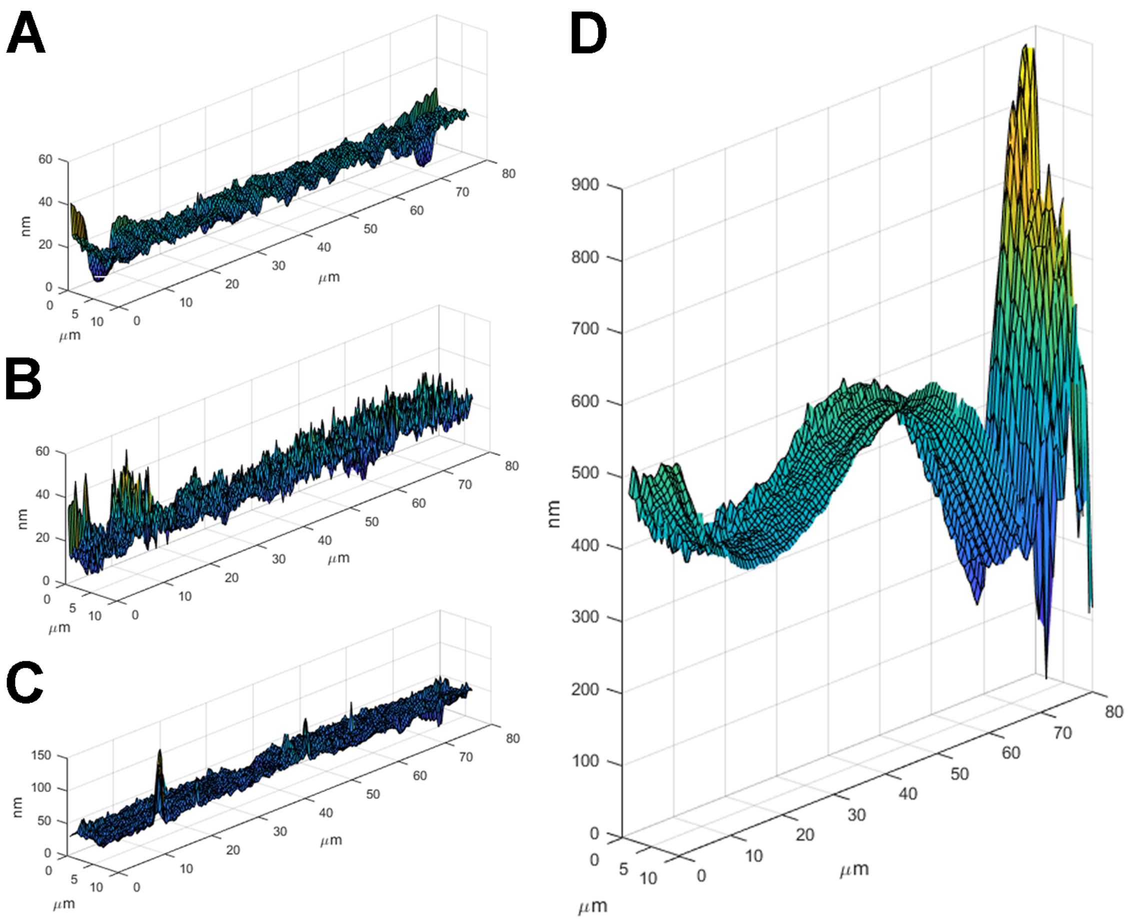

Supplementary table 1: Straightness of gap margins, standard deviation of the gap width, coefficient of variation of the gap width and gap closure rate

Results of Tukey multiple comparison testing between the 4 scratch methods (pipette 50 g, pipette 150g, magnet, cell exclusion insert) for the 4 parameters straightness of the gap margins, standard deviation of the gap width, coefficient of variation of the gap width and gap closure rate (slope).

Straightness of the Gap Margins			
	**Mean Diff.**	**95.00% CI of diff.**	**Adj. p value**
pipette 50 g vs. pipette 150 g	0.03	-0.0034 to 0.063	0.0946
pipette 50 g vs. magnet	0.1	0.067 to 0.13	**<0.0001**
pipette 50 g vs. insert	0.17	0.13 to 0.2	**<0.0001**
pipette 150 g vs. magnet	0.07	0.037 to 0.1	**<0.0001**
pipette 150 g vs. insert	0.14	0.1 to 0.17	**<0.0001**
magnet vs. insert	0.068	0.035 to 0.1	**<0.0001**
**Standard Deviation of the Gap Width**			
pipette 50 g vs. pipette 150 g	15	-4.7 to 36	0.1926
pipette 50 g vs. magnet	27	7.3 to 47	**0.0031**
pipette 50 g vs. insert	45	25 to 65	**<0.0001**
pipette 150 g vs. magnet	12	-8.4 to 32	0.4269
pipette 150 g vs. insert	30	9.4 to 50	**0.0013**
magnet vs. insert	18	-2.1 to 38	0.0956
**Coefficient of Variation of the Gap Width**			
pipette 50 g vs. pipette 150 g	2.6	0.97 to 4.2	**0.0008**
pipette 50 g vs. magnet	6.2	4.6 to 7.8	**<0.0001**
pipette 50 g vs. insert	4.7	3.1 to 6.3	**<0.0001**
pipette 150 g vs. magnet	3.6	2 to 5.2	**<0.0001**
pipette 150 g vs. insert	2.1	0.5 to 3.7	**0.0064**
magnet vs. insert	-1.5	-3.1 to 0.081	0.0674
**Gap Closure Rate (Slope)**			
pipette 50 g vs. pipette 150 g	-0.0002516	-0.0070 to 0.0065	0.9997
pipette 50 g vs. magnet	0.006026	-0.0006 to 0.0127	0.0891
pipette 50 g vs. insert	-0.01471	-0.0214 to -0.0081	**<0.0001**
pipette 150 g vs. magnet	0.006278	-0.0005 to 0.0131	0.0804
pipette 150 g vs. insert	-0.01446	-0.0213 to -0.0077	**<0.0001**
magnet vs. insert	-0.02074	-0.0274 to -0.0141	**<0.0001**

Supplementary table 2: Gap closure rate of chondrocytes and tenocytes

Descriptive statistics of the gap closure rate (slope) of chondrocytes and tenocytes and the results of Tukey multiple comparison testing between the 4 scratch methods (pipette 50 g, pipette 150g, magnet, cell exclusion insert) in chondrocyte and tenocyte monolayer cultures.

Chondrocytes				
	**pipette 50 g**	**pipette 150 g**	**magnet**	**Insert**
Mean	-0.03861	-0.03517	-0.04725	-0.02091
Std. Deviation	0.01183	0.009154	0.0085	0.01116
Std. Error of Mean	0.003414	0.002895	0.002454	0.003221
Lower 95% CI	-0.04613	-0.04172	-0.05265	-0.028
Upper 95% CI	-0.0311	-0.02862	-0.04185	-0.01383
Coefficient of variation	30.63%	26.03%	17.99%	53.35%
**Tukey multiple comparison**	**Mean Diff.**	**95.00% CI of diff.**		**Adj. p value**
pipette 50 g vs. pipette 150 g	-0.00344	-0.01329 to 0.006408		0.7968
pipette 50 g vs. magnet	0.008638	-0.0007514 to 0.01803		0.0827
pipette 50 g vs. ibidi	-0.0177	-0.02709 to -0.008309		**<0.0001**
pipette 150 g vs. magnet	0.01208	0.002231 to 0.02193		**0.0098**
pipette 150 g vs. ibidi	-0.01426	-0.02411 to -0.00441		**0.0015**
magnet vs. ibidi	-0.02634	-0.03573 to -0.01695		**<0.0001**
**Tenocytes**				
	**pipette 50 g**	**pipette 150 g**	**magnet**	**Insert**
Mean	-0.03383	-0.03664	-0.03725	-0.02211
Std. Deviation	0.006977	0.004641	0.01041	0.004322
Std. Error of Mean	0.002014	0.00134	0.003006	0.001248
Lower 95% CI	-0.03826	-0.03958	-0.04386	-0.02486
Upper 95% CI	-0.0294	-0.03369	-0.03063	-0.01936
Coefficient of variation	20.62%	12.67%	27.95%	19.55%
**Tukey multiple comparison**	**Mean Diff.**	**95.00% CI of diff.**		**Adj. p value**
pipette 50 g vs. pipette 150 g	0.002804	-0.006586 to 0.01219		0.8622
pipette 50 g vs. magnet	0.003414	-0.005976 to 0.0128		0.7765
pipette 50 g vs. ibidi	-0.01172	-0.02111 to -0.00233		**0.0083**
pipette 150 g vs. magnet	0.00061	-0.00878 to 0.01		0.9982
pipette 150 g vs. ibidi	-0.01452	-0.02391 to -0.005134		**0.0006**
magnet vs. ibidi	-0.01513	-0.02452 to -0.005744		**0.0003**

Supplementary table 3: Comparison of the number of dead cells between scratching techniques and cell exclusion technique

The number of dead cells counted for all three scratching techniques (50g, 150g, magnet) was higher compared to the cell culture insert for chondrocyte as well as tenocyte cultures.

**Comparison**	**Cell type**	**p-value**
50g versus insert	Chondrocytes	< 0.001
	Tenocytes	< 0.001
	Overall	< 0.001
150g versus insert	Chondrocytes	< 0.001
	Tenocytes	< 0.001
	Overall	< 0.001
magnet versus insert	Chondrocytes	< 0.001
	Tenocytes	< 0.001
	Overall	< 0.001

Supplementary table 4: Comparison of the number of dead cells between time points (0h, 8h, 24h) following "scratching" with all 4 methods (50g, 150g, cell culture insert and magnet) by cell type (chondrocytes and tenocytes)

As expected, the number of dead cells was highest at 0h after wounding. At the later time points (8h and 24h) the number of dead cells decreased, presumably because they detached leading to an even distribution of floating dead cells in the culture dish and thereby to a lower ratio of overall dead cells and dead cells in ROI(B) (region of main interest: contains cells which died in direct consequence to wounding).
The results were independent of the cell type (chondrocytes versus tenocytes). However, the scratches performed in the tenocyte cultures showed overall higher mean values and a wider range between minimum and maximum dead cell count.

Cell type	Wounding Technique	Time point	Mean	Standard Deviation	Minimum	Maximum
Chondrocytes	50g	0h	219.9	134.4	20.0	385.7
		8h	117.0	142.5	0	400
		24h	12.5	21.2	0	66.7
Chondrocytes	150g	0h	245.5	190.4	0	650.0
		8h	151.0	274.2	0	900.0
		24h	5.2	12.1	0	33.3
Chondrocytes	insert	0h	7.1	18.6	0	62.5
		8h	5.6	19.2	0	66.7
		24h	0	0	0	0
Chondrocytes	magnet	0h	533.9	385.9	161.1	1542.9
		8h	309.9	280.2	27.8	833.3
		24h	79.5	126.7	0	300
Tenocytes	50g	0h	300.1	322.1	0	1100.0
		8h	213.5	189.6	0	483.3
		24h	107.5	152.0	0	433.3
Tenocytes	150g	0h	622.8	478.3	60	1450.0
		8h	341.7	454.8	0	1500
		24h	68.1	108.3	0	300
Tenocytes	insert	0h	0	0	0	0
		8h	0.8	2.7	0	9.5
		24h	0	0	0	0
Tenocytes	magnet	0h	524.7	378.6	116.7	1520.0
		8h	679.9	579.7	50.0	1700.0
		24h	75.9	107.0	0	350.0
